# Case Report: 84 year-old woman with alien hand syndrome

**DOI:** 10.12688/f1000research.9096.1

**Published:** 2016-07-01

**Authors:** Ihtesham Aatif Qureshi, Daniel Korya, Darine Kassar, Mohammed Moussavi

**Affiliations:** 1 Texas Tech University of Health Sciences Center, El Paso, USA; 2 Stroke & Neurovascular Center at JFK Neuroscience Institute, Edison, USA

**Keywords:** Alien-Hand Syndrome, Hemorrhagic Stroke, involuntary movements, headache

## Abstract

**Background:**

Alien hand syndrome [AHS] is a rare and ill-defined neurological disorder. It produces complex, goal-directed motion of one hand that is involuntarily instigated. This syndrome characteristically arises after brain trauma, brain surgery, stroke or encephalitis. We describe a case of AHS in a patient who had a previous episode of subarachnoid hemorrhage affecting the left frontal lobe and corpus callosum.

**Case presentation:**

An 84-year-old woman presented to the emergency department complaining of headaches and several episodes of her left arm moving as if it was groping around trying to grab at her own body. A computed tomography scan of the head demonstrated an acute left superior frontal hemorrhage with compression of the corpus callosum. Transcranial Doppler report showed no significant abnormality in the insonated vessels. After being stabilized for the acute bleed, she was treated with clonazepam 0.5 mgat night for the uncontrolled hand movements. Her movements resolved by her next month follow up. The diagnosis of AHS was made based on her clinical presentation, characterization of the movement and localization correlating with findings in neuroimaging.

**Conclusion:**

We document a rare neurologic disorder seen in patients presenting with a history of previous strokes and a typical description of involuntary and unintentional, uncontrolled unilateral arm movements with repetitive grasping. The present case has a combination of frontal and callosal lesions.  These findings appear to support a potential destruction leading to the rare syndrome.

## Introduction

Alien hand syndrome [AHS] is a loosely defined collection of symptoms characterized by involuntary movement of an upper limb in concurrence with the experience of separation from or exemplification of the actions of the limb itself. AHS was primarily used to label cases involving a disconnection of the hemispheres associated with a lesion of the corpus callosum ^1^. There are three main types of AHS: frontal AHS (associated with lesions of the medial frontal cortex and characterized by reflex grasping and compulsive manipulation of tools); callosal AHS (associated with lesions of the corpus callosum and characterized by inter-manual conflict); and posterior cortical AHS (associated with parietal, occipital, thalamic structural lesions, cortico-basal degeneration, and characterized by sensory ataxia and feelings of estrangement of the upper limb) ^2, 3^.

In the present case, we describe a patient who presented with subarachnoid hemorrhage involving the left medial aspect (or parasagittal region) of the frontal lobe and caused the patient to present with headache and unconscious left hand movements consistent with AHS. We supplement the case presentation with a literature review of radiographic findings, past medical history and presenting symptoms of AHS.

## Case presentation

An 84-year-old African-American right hand dominant woman presented to the emergency department complaining of headache and episodes of uncontrollable left hand movements. She described the episodes wherein her left arm moved uncontrollably as if it was groping around trying to grab herself on her body. The patient explained that while asleep she felt that “someone is trying to grab meas if someone is in bed with me”. At times, she felt the need to talk to her hand or yell at it in order to command it to stop these embarrassing movements. These movements occurred while she was attempting to eat, watch television and during use of the toilet. The patient was evidently very distressed by these events and thought that her arm was “possessed by the Devil”.

Her past medical history included chronic anemia, hypertension, previous intracranial hemorrhage, glaucoma, breast cancer (in remission) and cataracts. She was surgically treated for her breast cancer with lumpectomy and had bilateral cataract surgery in the past. Her family history consisted of diabetes, hypertension, migraine headaches and thyroid disease. She denied previous smoking or drinking. Her medications included anastrozole, latanoprost, amlodipine, pravastatin and multivitamin. She had no allergies.

On physical examination, her vitals were pulse 72/min, blood pressure was 140/70 mm Hg, weight was 130 lbs and height was 62 inches. She was comfortably seated and pleasant. No carotid bruits were appreciated. Heart rate and rhythm were regular. Normal pulses with no edema, cyanosis or clubbing noted on extremities. She was alert and oriented with fluent speech and no dysarthria. Cranial nerves II-XII were tested and normal. Fundi were benign with flat, well-marginated discs. There were no hemorrhages or exudates. The patient had intact visual fields to confrontation. Pupils were equal, round and reactive to light and accommodation. There was no afferent pupillary defect. Extra-ocular movements were normal. Facial motor and sensation were symmetric. Palate was symmetric on phonation and tongue protruded to the midline. Sternocleidomastoids were both full in strength.

On motor examination, she had strength of 5/5 on left arm and leg, whereas on the right side there was a considerable weakness with 2/5 strength on right arm and 3/5 on right leg. The deep tendon reflexes were brisk on the right. Plantar reflexes were down-going on the left and equivocal on the right. Coordination was worse on the left upper extremity with worse finger tapping. Sensory examination for pin-prick, temperature and vibration was symmetrically intact. The patient was able to stand and walk without assistance. Romberg test was negative.

**Figure 1.**
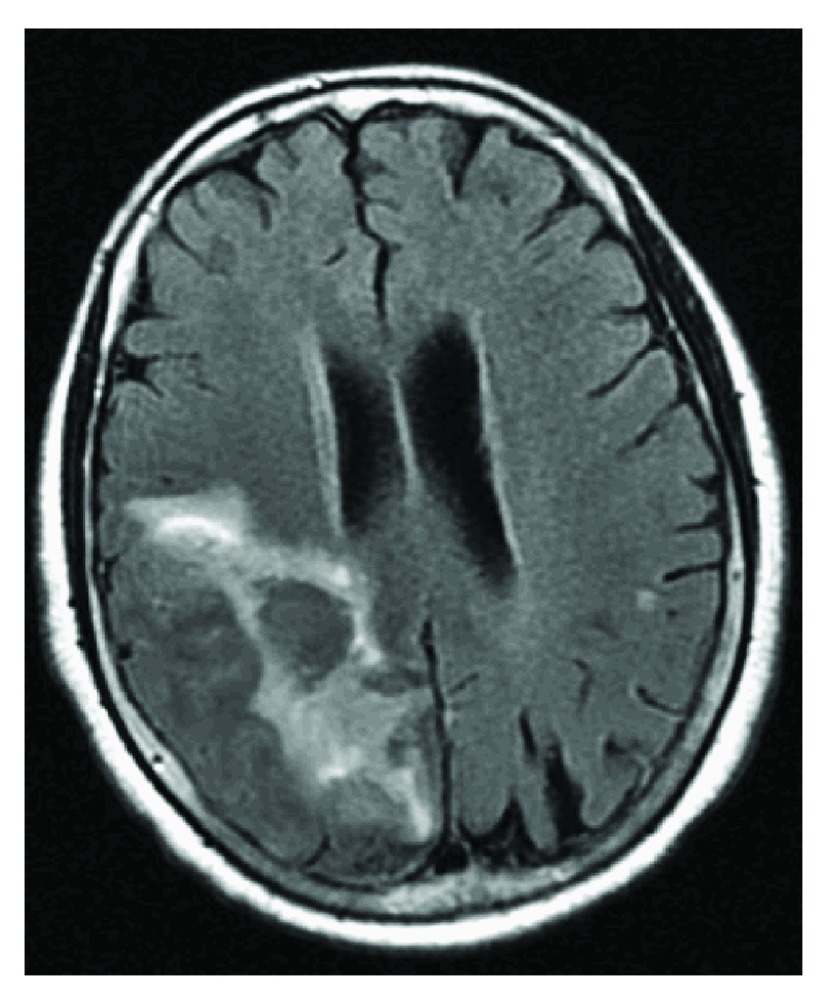
MRI-T2 Flair showing hyperintensity extending from posterior frontal, parietal causing mass effect compressing the midline and posterior corpus callosum.

**Figure 2.**
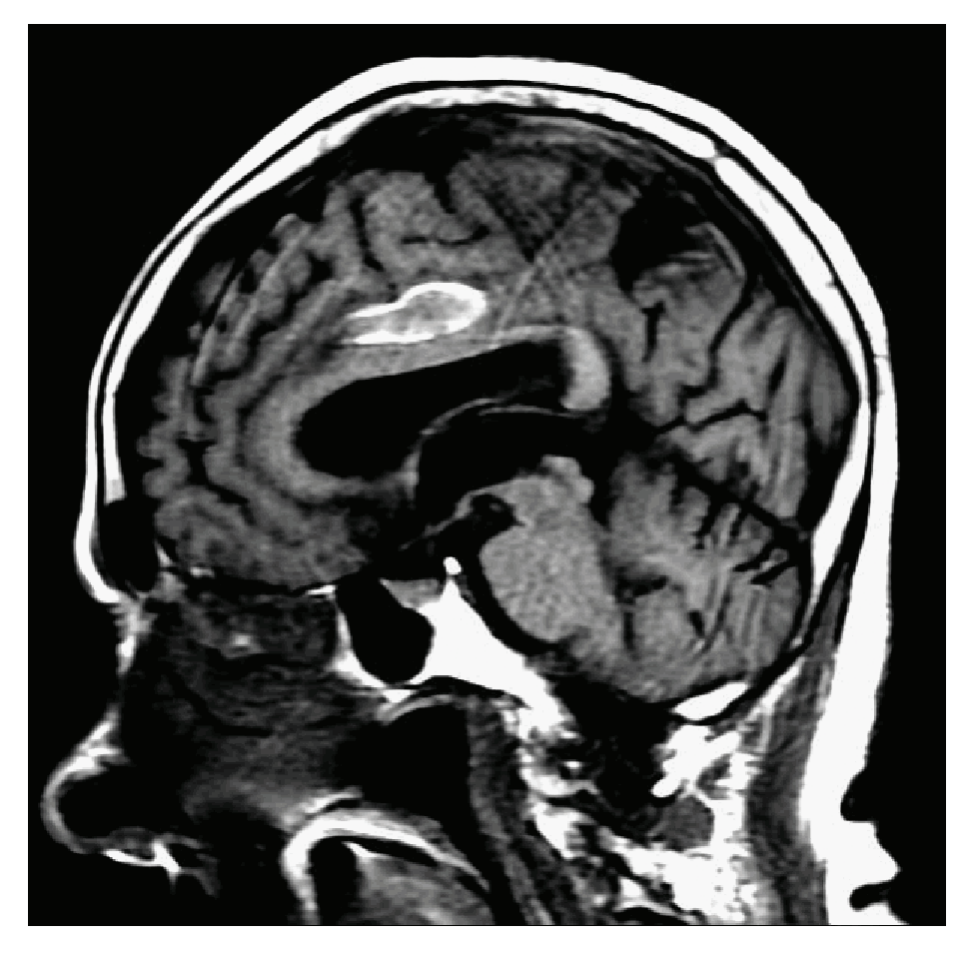
Sagittal MRI T2-FLAIR showing hyperintensity in the parasagittal region compressing corpus callosum and extending into the left parasagittal region.

A computed tomography (CT) scan of the head was performed and showed an acute left frontal, parasagittal hematoma measuring 3 cm. Radiographic findings with magnetic resonance imaging (MRI) of the brain with and without contrast on axial MRI-T2 FLAIR ( Figure 1) and sagittal MRI-T2 FLAIR ( Figure 2) images were acquired.

A Transcranial Doppler (TCD) study was done due to the clinical history of subarachnoid hemorrhage, and showed a normal direction of blood flow with mean flow velocities and spectral waveform within normal limits in all insonated segments of circle of Willis. The final impression was suggestive of no significant abnormality in the insonated vessels.

The patient was observed in the neurocritical care unit and then transferred to the stroke unit. After being stabilized, she was discharged to follow up within the neurovascular clinic. She presented to the clinic two weeks later with continued complaints of the left hand movements. At this point, the patient was expressing significant frustration and asked for assistance for these “Devilish movements”. After complete examination, the patient was recommended clonazepam 0.5 mg at night for the involuntary movements, was advised to avoid anti-platelet agents, and only take acetaminophen 500 mg for headaches. The patient was advised to return to the clinic after one month to determine if the medication was helping. At her one-month follow-up, the patient was pleased to report that her symptoms had decreased significantly and had last occurred about one week prior to her clinic visit.

## Discussion

AHS is a rare neurological disorder, typically seen during or after a vascular lesion of the brain. There are mainly three types of AHS. Two are frontal varieties, of which one is linked to lesions of the language dominant medial frontal cortex and the anterior corpus callosum affecting dominant hand, and the other involves the corpus callosum alone and affects the non-dominant hand ^4– 7^.The callosal-frontal alien hand variant usually results in more grasping activities and compulsive manipulations and the callosal AHS presents predominantly with inter-manual conflict ^4^. The presenting case had a combination of the two frontal variants. Frontal variant lesion was caused by intracerebral and subarachnoid hemorrhage involving the territory of the anterior cerebral artery (ACA) affecting both the left frontal lobe and the corpus callosum.

Premotor cortex, motor cortex and anterior two-thirds of the corpus callosum is supplied by ACA. Inter-hemispheric connections may be interrupted by occlusion of ACA. Proximal occlusions may result in motor weakness of the contralateral limbs along with compulsive reflex movements such as grasp reflex ^8^.

Patient with left hemispheric brain tumor invading the corpus callosum resulted in involuntary grasping and dropping of objects with the contralateral hand as described by Van Vleuten ^9^. The term “alien hand syndrome” involves repetitive involuntary goal oriented limb movements acting opposite to the individual’s objective ^10^. This type of movement was first reported by Van Vleuten.

In our case, the patient’s left hand was not only apraxic, but also accomplished distinctly improper actions, such as touching her right hand instead of nose, regardless of her understanding of the command, and failing to move when instructed.

The term “ *la main etrangere“* was coined by Brion and Jedynak ^11^ describes failure to identify self-ownership of the limb or lack of self-care over the goal-directed limb movements among patients with callosal tumors. A milder version of this intermanual conflict was seen among patients with surgical callosal lesions. This was termed as “alien hand” by Bogen ^12^, a translation of Brion and Jedynak’s *la main etrangere*.

In the case presented, the patient’s left arm was affected due to AHS, and the involuntary movement initially occurred daily, but later became less frequent. In a literature review, decrease in symptoms occurred in 68% of patients, whereas symptoms persisted in 32% ^13^.

Overall, the findings of this case represent frontal variants of previously described patterns causing AHS. This occurrence of the symptoms descriptive of the syndrome is seen after the affected corpus callosum’s communicating fibers disconnected from the functional left cortical region.

## Conclusion

The development of AHS may be dependent on an injury pattern that first causes dysfunction of the association motor cortex of the right side and a subsequent lack of communication with the dominant left side, which would otherwise provide orders to the damaged non-dominant area. The patient’s symptoms were almost completely resolved with the use of clonazepam, but most patients improve spontaneously. As more reports emerge about this rare phenomenon, a greater understanding of the underlying mechanisms and causes of the dysfunction can be expected.

## Patient consent

Informed written consent for publication of clinical details was obtained from the patient.
