# Supplementary material for: Case Report: 84 year-old woman with alien hand syndrome
Source: F1000Res. 2016 Jul 1;5:1564. [Version 1] doi: 10.12688/f1000research.9096.1 (PMC5155497; doi:10.12688/f1000research.9096.1)
Supplement: Peer review history

# Peer review: Referee response for version 1

**DOI:** 10.5256/f1000research.9790.r18081

**Published:** 2016-12-09

**Authors:** Mary Ann Thenganatt

**Affiliations:** 1 Department of Neurology, Perelman School of Medicine, University of Pennsylvania, Philadelphia, PA, USA

This is an interesting report of a case of alien hand syndrome. It is clearly written and provides background on the history and classification of alien hand variants. The authors provide a detailed clinical description of this syndrome caused by a vascular insult,  as well as discuss diagnostic studies, treatment and outcome of this case.

I have read this submission. I believe that I have an appropriate level of expertise to confirm that it is of an acceptable scientific standard.

# Peer review: Referee response for version 1

**DOI:** 10.5256/f1000research.9790.r16625

**Published:** 2016-10-10

**Authors:** Burak Yulug, Lütfü Hanoglu

**Affiliations:** 1 Department of Neurology, University of Istanbul-Medipol, Istanbul, Turkey

The authors well described an unusual case of AHS in CVD. However, I think that the following major comments would help to improve the impact of the manuscript:

1. The authors should clearly explain what they mean by stabilized. Are the AHS symptoms improved or do they mean the stabilization of neurovascular symptomatology only?
2. Acute CT findings including the CT figure showing the acute cerebrovascular pathology should be added. 
3. They should describe the frequency of similar combined cerebrovascular cases in the literature.  
4. What about the therapeutic effects of Clonazepam in the literature? This also should be added in the manuscript. 
5. It is not clear how a left acute superior frontal pathology can lead to dysfunction of the right association cortex that secondary leads to a dysregulation of communication with the left side (Conclusion section). I think they should clearly define the hypothesized pathophysiology and include an illustration defining the disturbed communication and functional interaction in details. 

We have read this submission. We believe that we have an appropriate level of expertise to confirm that it is of an acceptable scientific standard, however we have significant reservations, as outlined above.

# Peer review: Referee response for version 1

**DOI:** 10.5256/f1000research.9790.r14782

**Published:** 2016-07-12

**Authors:** Nizar Souayah

**Affiliations:** 1 Department of Neurology and Neurosciences, New Jersey Medical School, Rutgers, The State University of New Jersey, Newark, NJ, USA

This is an interesting case report of a patient who developed an AHS with involuntary movement after a stroke  in the frontal and callosal area.  The report was well written and supported by literature.

I have read this submission. I believe that I have an appropriate level of expertise to confirm that it is of an acceptable scientific standard.
